# *tert*-Butyl 4-{5-[3-(tri­fluoro­meth­oxy)phen­yl]-1,2,4-oxa­diazol-3-yl}piperazine-1-carboxyl­ate

**DOI:** 10.1107/S1600536813010131

**Published:** 2013-04-20

**Authors:** Swamy Sreenivasa, Karikere Ekanna ManojKumar, Arakyathanahalli Kempaiah, Parameshwar Adimoole Suchetan, Bandrehalli Siddagangaiah Palakshamurthy

**Affiliations:** a Department of Studies and Research in Chemistry, Tumkur University, Tumkur, Karnataka 572 103, India; b Department of Physics, Governament First Grade College K.R. Pete, Karnataka 571 426, India; c Department of Studies and Research in Chemistry, U.C.S., Tumkur University, Tumkur, Karnataka 572 103, India; d Department of Studies and Research in Physics, U.C.S., Tumkur University, Tumkur, Karnataka 572 103, India

## Abstract

In the title compound, C_18_H_21_F_3_N_4_O_4_, the piperazine ring adopts a chair conformation and the dihedral angle between the oxa­diazole and benzene rings is 6.45 (14)°. The C atoms and their attached H atoms in the piperazine ring are disordered, with site-occupation factors of 0.576 (12) and 0.424 (12). In the crystal, mol­ecules are linked through weak C—H⋯O inter­actions, generating an *R*_2_^2^(12) motif. Further, secondary C—H⋯O inter­molecular inter­actions link the mol­ecules into *C*(6) chains along [100].

## Related literature

For the synthesis and biological activity of 1,2,4-oxa­diazo­les, see: Chimirri *et al.* (1996 ▶); Nicolaides *et al.* (1998 ▶); Kemnitzer *et al.* (2009 ▶).
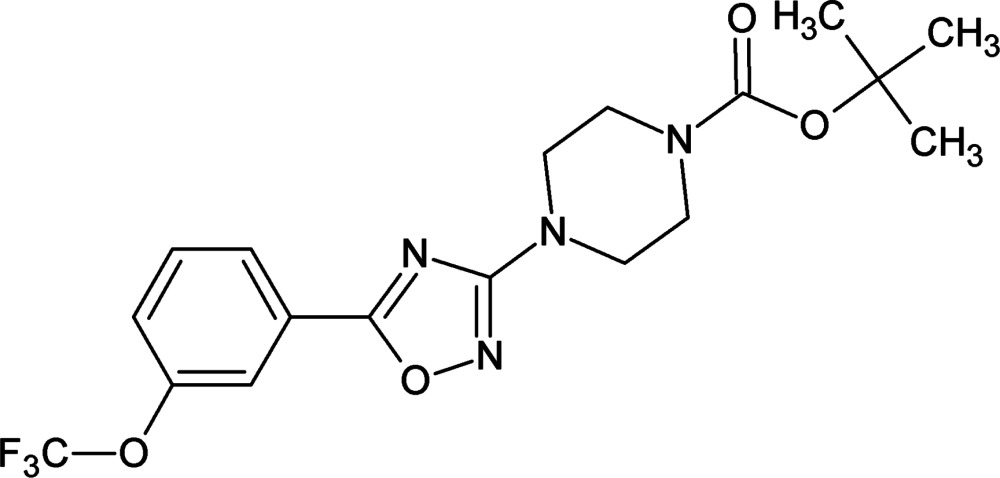


## Experimental

### 

#### Crystal data

C_18_H_21_F_3_N_4_O_4_
*M* *_r_* = 414.39
Triclinic, 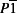

*a* = 5.773 (2) Å
*b* = 11.168 (5) Å
*c* = 15.991 (7) Å
α = 96.092 (16)°
β = 100.316 (14)°
γ = 91.333 (14)°
*V* = 1007.7 (8) Å^3^
*Z* = 2
Mo *K*α radiation
μ = 0.12 mm^−1^
*T* = 300 K
0.28 × 0.24 × 0.18 mm

#### Data collection

Bruker SMART X2S diffractometer
Absorption correction: multi-scan (*SADABS*; Bruker, 2009 ▶) *T*_min_ = 0.968, *T*_max_ = 0.980
7493 measured reflections
3521 independent reflections
2233 reflections with *I* > 2σ(*I*)
*R*_int_ = 0.040

#### Refinement

*R*[*F* ^2^ > 2σ(*F* ^2^)] = 0.062
*wR*(*F* ^2^) = 0.207
*S* = 1.10
3521 reflections
303 parameters
H-atom parameters constrained
Δρ_max_ = 0.40 e Å^−3^
Δρ_min_ = −0.23 e Å^−3^

### 

Data collection: *APEX2* (Bruker, 2009 ▶); cell refinement: *APEX2* and *SAINT-Plus* (Bruker, 2009 ▶); data reduction: *SAINT-Plus* and *XPREP* (Bruker, 2009 ▶); program(s) used to solve structure: *SHELXS97* (Sheldrick, 2008 ▶); program(s) used to refine structure: *SHELXL97* (Sheldrick, 2008 ▶); molecular graphics: *Mercury* (Macrae *et al.*, 2008 ▶); software used to prepare material for publication: *SHELXL97*.

## Supplementary Material

Crystal structure: contains datablock(s) I, global. DOI: 10.1107/S1600536813010131/bt6899sup1.cif

Structure factors: contains datablock(s) I. DOI: 10.1107/S1600536813010131/bt6899Isup2.hkl

Supplementary material file. DOI: 10.1107/S1600536813010131/bt6899Isup3.cml

Additional supplementary materials:  crystallographic information; 3D view; checkCIF report

## Figures and Tables

**Table 1:** Hydrogen-bond geometry (Å, °)

*D*—H⋯*A*	*D*—H	H⋯*A*	*D*⋯*A*	*D*—H⋯*A*
C17—H17*A*⋯O4^i^	0.96	2.56	3.393 (3)	145
C10*A*—H10*C*⋯O4^ii^	0.97	2.46	3.413 (10)	167

Symmetry codes: (i) 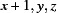; (ii) 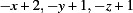.

**Fig. 1.**
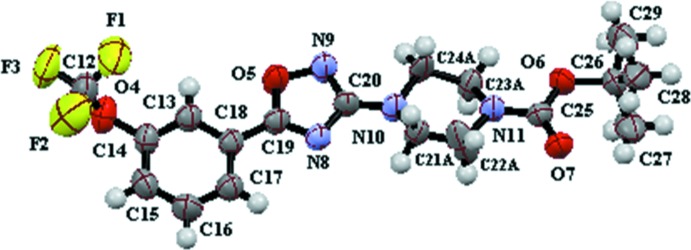
Molecular structure of the title compound, showing displacement ellipsoids drawn at the 50% probability level.

**Fig. 2.**
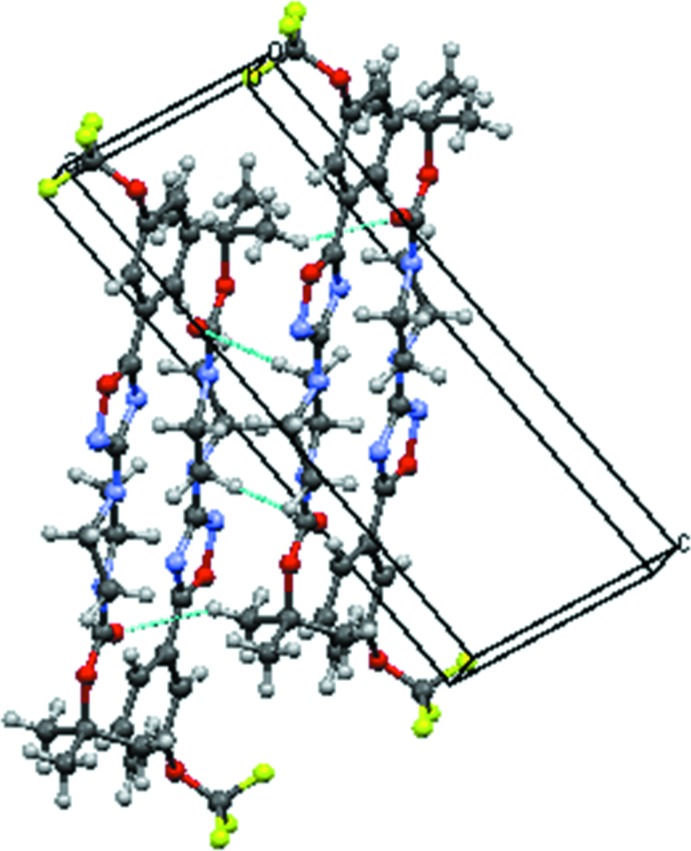
Packing of molecules in the crystal structure along a axis.

## supplementary crystallographic information

### Comment

1,2,4-Oxadiazoles exhibit diverse biological activities (Chimirri *et al.*, 1996). They have been described as bio-isosteres for amides and esters. Due to increased hydrolytic and metabolic stabilities of the oxadiazole ring, improved pharmacokinetic and *in vivo* performance are often observed, which makes these heterocycles an important structural moiety for the pharmaceutical industry (Nicolaides *et al.*, 1998). As a consequence of these, oxadiazoles have often been the target of numerous drug discovery programs as anti-inflammatory agents, anti-tumor agents, potential anticancer agents, Histamine H3 receptor antagonists as potent inhibitors of MIF biological function, and bell-tryptase inhibitors, In addition to these, 1,2,4-oxadiazoles are widely used as hydrolysis resisting amide bioisosteres in the development of peptidomimetics (Kemnitzer *et al.*,2009). Also, oxadiazoles exhibit wide range of antibacterial, antifungal and activities against Gram-positive and Gram-negative bacteria. Keeping this in mind, the crystal structure of the title compound was determined.

In the crystal structure of the title compound, C_18_H_21_F_3_N_4_O_4_, the piperazine ring adopts chair conformation, and the molecule is almost planar with the dihedral angle between the oxadiazole and the benzene ring is 6.45 (14)*^o^*. In the structure, the molecules are linked through weak C10A—H10C···O4 interactions generating a *R*_2_^2^(12) motif. Further, C17—H17A···O4 intermolecular interactions link the molecules into C(6) chains along [100].

### Experimental

To a solution of 1-*N*-carbidamide (2.14 mmol) in 5 ml *N*,*N*-dimethylformamide was added 3-trifluoromethoxy benzoic acid (2.36 mmol) and propylphosphonic anhydride (4.72 mmol). The reaction mixture was heated at 150°C for 12 h (reaction was monitored by TLC). The reaction mixture was poured to ice cold water. The solid obtained was filtered and washed with water. The crude product was purified by column chromatography using pet ether-ethyl acetate as the eluent.

Colourless prisms were obtained from slow evaporation of the solution of the compound in a mixture of pet ether and ethyl acetate (1:2).

### Refinement

All H atoms were positioned geometrically, with C—H = 0.93 Å for aromatic H, C—H = 0.97 Å for methylene H and C —H = 0.96 Å for methyl H, and refined using a riding model with *U*_iso_(H) = 1.5Ueq(C) for methyl H and *U*_iso_(H) = 1.2Ueq(C) for all other H.

The C10, C11 C12 and C13 carbon atoms of a piperazine ring in the molecules were disordered over two sites and refined with site occupancy factors 0.576 (12):0.424 (12).

### Crystal data

**Table:**

C_18_H_21_F_3_N_4_O_4_	*F*(000) = 432
*M**_r_* = 414.39	prism
Triclinic, *P*1	*D*_x_ = 1.366 Mg m^−^^3^
Hall symbol: -P 1	Melting point: 435 K
*a* = 5.773 (2) Å	Mo *K*α radiation, λ = 0.71073 Å
*b* = 11.168 (5) Å	Cell parameters from 3543 reflections
*c* = 15.991 (7) Å	θ = 1.8–25°
α = 96.092 (16)°	µ = 0.12 mm^−^^1^
β = 100.316 (14)°	*T* = 300 K
γ = 91.333 (14)°	Prism, colourless
*V* = 1007.7 (8) Å^3^	0.28 × 0.24 × 0.18 mm
*Z* = 2	

### Data collection

**Table:**

Bruker SMART X2S diffractometer	3521 independent reflections
Radiation source: fine-focus sealed tube	2233 reflections with *I* > 2σ(*I*)
Graphite monochromator	*R*_int_ = 0.040
Detector resolution: 1.20 pixels mm^-1^	θ_max_ = 25.0°, θ_min_ = 1.8°
phi and ω scans	*h* = −6→6
Absorption correction: multi-scan (*SADABS*; Bruker, 2009)	*k* = −13→13
*T*_min_ = 0.968, *T*_max_ = 0.980	*l* = −17→18
7493 measured reflections	

### Refinement

**Table:**

Refinement on *F*^2^	Secondary atom site location: difference Fourier map
Least-squares matrix: full	Hydrogen site location: inferred from neighbouring sites
*R*[*F*^2^ > 2σ(*F*^2^)] = 0.062	H-atom parameters constrained
*wR*(*F*^2^) = 0.207	*w* = 1/[σ^2^(*F*_o_^2^) + (0.1182*P*)^2^ + 0.0138*P*] where *P* = (*F*_o_^2^ + 2*F*_c_^2^)/3
*S* = 1.10	(Δ/σ)_max_ < 0.001
3521 reflections	Δρ_max_ = 0.40 e Å^−^^3^
303 parameters	Δρ_min_ = −0.23 e Å^−^^3^
0 restraints	Extinction correction: *SHELXL97* (Sheldrick, 2008), Fc^*^=kFc[1+0.001xFc^2^λ^3^/sin(2θ)]^-1/4^
0 constraints	Extinction coefficient: 0.183 (18)
Primary atom site location: structure-invariant direct methods	

### Special details

**Table:**

Geometry. All e.s.d.'s (except the e.s.d. in the dihedral angle between two l.s. planes) are estimated using the full covariance matrix. The cell e.s.d.'s are taken into account individually in the estimation of e.s.d.'s in distances, angles and torsion angles; correlations between e.s.d.'s in cell parameters are only used when they are defined by crystal symmetry. An approximate (isotropic) treatment of cell e.s.d.'s is used for estimating e.s.d.'s involving l.s. planes.
Refinement. Refinement of *F*^2^ against ALL reflections. The weighted *R*-factor *wR* and goodness of fit *S* are based on *F*^2^, conventional *R*-factors *R* are based on *F*, with *F* set to zero for negative *F*^2^. The threshold expression of *F*^2^ > σ(*F*^2^) is used only for calculating *R*-factors(gt) *etc*. and is not relevant to the choice of reflections for refinement. *R*-factors based on *F*^2^ are statistically about twice as large as those based on *F*, and *R*- factors based on ALL data will be even larger.

### Fractional atomic coordinates and isotropic or equivalent isotropic displacement parameters (Å^2^)

**Table:**

	*x*	*y*	*z*	*U*_iso_*/*U*_eq_	Occ. (<1)
F1	0.1439 (5)	−0.1754 (2)	0.02278 (16)	0.1429 (9)	
F2	−0.1943 (5)	−0.1403 (2)	−0.03649 (15)	0.1536 (11)	
F3	−0.1037 (5)	−0.31943 (19)	−0.01851 (15)	0.1365 (9)	
O3	1.2946 (3)	0.41202 (15)	0.72076 (12)	0.0732 (6)	
N1	0.3871 (4)	0.16222 (17)	0.37686 (14)	0.0639 (6)	
O1	−0.1462 (4)	−0.20090 (16)	0.09264 (14)	0.0872 (7)	
O4	1.0601 (3)	0.56341 (15)	0.68027 (12)	0.0729 (6)	
N4	0.9956 (4)	0.37620 (18)	0.61086 (15)	0.0775 (8)	
N3	0.7139 (4)	0.20950 (18)	0.49149 (15)	0.0748 (7)	
C8	0.3252 (4)	0.0647 (2)	0.32603 (17)	0.0626 (7)	
O2	0.4744 (3)	−0.02502 (15)	0.33963 (13)	0.0842 (7)	
C17	1.5616 (5)	0.5904 (3)	0.7642 (2)	0.0842 (9)	
H17A	1.6533	0.5603	0.7226	0.126*	
H17B	1.6630	0.6351	0.8120	0.126*	
H17C	1.4451	0.6421	0.7387	0.126*	
C15	1.4403 (4)	0.4856 (2)	0.79455 (18)	0.0675 (8)	
C14	1.1144 (4)	0.4601 (2)	0.67156 (17)	0.0598 (7)	
C13B	0.926 (4)	0.1677 (18)	0.5545 (17)	0.120 (9)	0.424 (12)
H13A	0.8738	0.1465	0.6056	0.144*	0.424 (12)
H13B	0.9903	0.0971	0.5279	0.144*	0.424 (12)
C12B	1.099 (2)	0.2613 (8)	0.5762 (10)	0.086 (4)	0.424 (12)
H12A	1.1640	0.2757	0.5261	0.103*	0.424 (12)
H12B	1.2261	0.2381	0.6191	0.103*	0.424 (12)
C10B	0.630 (2)	0.3260 (9)	0.5233 (12)	0.088 (4)	0.424 (12)
H10A	0.5704	0.3662	0.4734	0.105*	0.424 (12)
H10B	0.4948	0.3079	0.5490	0.105*	0.424 (12)
C11B	0.752 (2)	0.3985 (9)	0.5728 (7)	0.058 (3)	0.424 (12)
H11A	0.6698	0.4182	0.6199	0.070*	0.424 (12)
H11B	0.7584	0.4709	0.5445	0.070*	0.424 (12)
C12A	1.0048 (16)	0.2456 (4)	0.6218 (5)	0.069 (2)	0.576 (12)
H12C	0.8964	0.2242	0.6582	0.083*	0.576 (12)
H12D	1.1626	0.2261	0.6476	0.083*	0.576 (12)
C10A	0.7026 (16)	0.3384 (6)	0.4823 (4)	0.064 (2)	0.576 (12)
H10C	0.7741	0.3522	0.4335	0.076*	0.576 (12)
H10D	0.5374	0.3557	0.4674	0.076*	0.576 (12)
C11A	0.798 (3)	0.4188 (9)	0.5456 (10)	0.118 (5)	0.576 (12)
H11C	0.6745	0.4481	0.5754	0.142*	0.576 (12)
H11D	0.8589	0.4866	0.5216	0.142*	0.576 (12)
C13A	0.935 (2)	0.1786 (10)	0.5329 (10)	0.079 (3)	0.576 (12)
H13C	1.0519	0.1964	0.4990	0.095*	0.576 (12)
H13D	0.9323	0.0925	0.5371	0.095*	0.576 (12)
C9	0.5908 (4)	0.1308 (2)	0.42731 (17)	0.0650 (7)	
C7	0.1175 (4)	0.0417 (2)	0.25840 (16)	0.0602 (7)	
C2	0.0872 (5)	−0.0670 (2)	0.20452 (17)	0.0643 (7)	
H2	0.2013	−0.1244	0.2102	0.077*	
C3	−0.1129 (5)	−0.0876 (2)	0.14335 (17)	0.0680 (7)	
C1	−0.0818 (7)	−0.2073 (3)	0.0164 (2)	0.0931 (10)	
C16	1.2901 (5)	0.5271 (3)	0.8599 (2)	0.0908 (10)	
H16A	1.1790	0.5829	0.8365	0.136*	
H16B	1.3893	0.5660	0.9106	0.136*	
H16C	1.2069	0.4588	0.8739	0.136*	
C18	1.6170 (5)	0.3967 (3)	0.8286 (2)	0.1016 (11)	
H18A	1.5353	0.3288	0.8440	0.152*	
H18B	1.7219	0.4349	0.8782	0.152*	
H18C	1.7059	0.3698	0.7854	0.152*	
N2	0.6565 (4)	0.02094 (19)	0.40855 (17)	0.0873 (8)	
C6	−0.0521 (5)	0.1271 (2)	0.24771 (19)	0.0719 (8)	
H6	−0.0305	0.2007	0.2818	0.086*	
C5	−0.2519 (5)	0.1028 (3)	0.1868 (2)	0.0873 (9)	
H5	−0.3673	0.1597	0.1813	0.105*	
C4	−0.2857 (5)	−0.0051 (3)	0.13307 (19)	0.0802 (9)	
H4	−0.4209	−0.0208	0.0915	0.096*	

### Atomic displacement parameters (Å^2^)

**Table:**

	*U*^11^	*U*^22^	*U*^33^	*U*^12^	*U*^13^	*U*^23^
F1	0.1346 (19)	0.166 (2)	0.1214 (19)	−0.0219 (17)	0.0325 (16)	−0.0246 (16)
F2	0.213 (3)	0.157 (2)	0.0844 (15)	0.064 (2)	−0.0016 (16)	0.0215 (14)
F3	0.176 (2)	0.0978 (15)	0.1147 (18)	−0.0047 (14)	0.0031 (15)	−0.0393 (12)
O3	0.0603 (10)	0.0590 (10)	0.0896 (14)	0.0103 (8)	−0.0093 (10)	−0.0032 (9)
N1	0.0682 (13)	0.0509 (11)	0.0667 (14)	0.0126 (9)	−0.0016 (11)	0.0007 (9)
O1	0.1116 (16)	0.0655 (12)	0.0754 (15)	−0.0164 (11)	0.0025 (12)	−0.0052 (10)
O4	0.0692 (11)	0.0581 (11)	0.0865 (14)	0.0138 (9)	0.0067 (10)	−0.0043 (9)
N4	0.0780 (14)	0.0522 (12)	0.0861 (17)	0.0204 (10)	−0.0215 (12)	−0.0078 (11)
N3	0.0790 (14)	0.0505 (12)	0.0815 (16)	0.0204 (10)	−0.0169 (12)	−0.0041 (10)
C8	0.0711 (16)	0.0492 (13)	0.0654 (16)	0.0117 (11)	0.0056 (13)	0.0056 (11)
O2	0.0890 (13)	0.0546 (10)	0.0924 (15)	0.0194 (9)	−0.0193 (11)	−0.0095 (9)
C17	0.0629 (16)	0.087 (2)	0.101 (2)	−0.0115 (15)	0.0223 (16)	−0.0084 (17)
C15	0.0530 (13)	0.0693 (16)	0.0728 (18)	0.0025 (12)	−0.0011 (13)	−0.0046 (13)
C14	0.0553 (14)	0.0519 (14)	0.0706 (17)	0.0082 (11)	0.0102 (12)	0.0006 (11)
C13B	0.091 (9)	0.085 (9)	0.16 (2)	0.005 (6)	−0.064 (10)	0.038 (9)
C12B	0.089 (6)	0.061 (4)	0.095 (8)	0.033 (4)	−0.008 (6)	−0.013 (5)
C10B	0.074 (6)	0.074 (6)	0.104 (10)	0.032 (5)	−0.013 (6)	0.000 (7)
C11B	0.077 (5)	0.030 (4)	0.059 (5)	0.024 (4)	−0.005 (4)	−0.007 (4)
C12A	0.089 (4)	0.053 (3)	0.056 (4)	0.014 (3)	−0.011 (3)	0.004 (3)
C10A	0.080 (5)	0.053 (3)	0.051 (4)	0.023 (3)	−0.008 (3)	0.000 (3)
C11A	0.108 (7)	0.062 (4)	0.160 (11)	−0.005 (4)	−0.056 (7)	0.048 (6)
C13A	0.098 (6)	0.045 (4)	0.077 (5)	0.037 (4)	−0.024 (4)	−0.014 (4)
C9	0.0667 (15)	0.0521 (13)	0.0700 (17)	0.0135 (11)	−0.0035 (13)	0.0037 (11)
C7	0.0646 (14)	0.0509 (13)	0.0630 (16)	0.0045 (11)	0.0064 (13)	0.0047 (11)
C2	0.0717 (16)	0.0513 (13)	0.0677 (17)	0.0062 (11)	0.0052 (14)	0.0081 (11)
C3	0.0798 (17)	0.0557 (14)	0.0625 (17)	−0.0042 (12)	0.0012 (14)	0.0009 (11)
C1	0.105 (3)	0.079 (2)	0.080 (2)	0.0066 (18)	−0.012 (2)	−0.0086 (17)
C16	0.0748 (18)	0.122 (3)	0.076 (2)	−0.0063 (17)	0.0193 (16)	0.0046 (18)
C18	0.0774 (19)	0.094 (2)	0.120 (3)	0.0150 (16)	−0.0166 (19)	0.007 (2)
N2	0.0873 (15)	0.0587 (13)	0.0973 (19)	0.0220 (11)	−0.0248 (14)	−0.0095 (12)
C6	0.0728 (17)	0.0633 (15)	0.0734 (18)	0.0102 (13)	0.0005 (14)	−0.0005 (13)
C5	0.0749 (18)	0.0794 (19)	0.100 (2)	0.0201 (15)	−0.0017 (18)	0.0023 (17)
C4	0.0660 (16)	0.086 (2)	0.080 (2)	−0.0004 (15)	−0.0055 (15)	0.0018 (15)

### Geometric parameters (Å, º)

**Table:**

F1—C1	1.326 (4)	C12B—H12B	0.9700
F2—C1	1.287 (4)	C10B—C11B	1.192 (16)
F3—C1	1.309 (3)	C10B—H10A	0.9700
O3—C14	1.346 (3)	C10B—H10B	0.9700
O3—C15	1.477 (3)	C11B—H11A	0.9700
N1—C8	1.290 (3)	C11B—H11B	0.9700
N1—C9	1.377 (3)	C12A—C13A	1.517 (15)
O1—C1	1.333 (4)	C12A—H12C	0.9700
O1—C3	1.418 (3)	C12A—H12D	0.9700
O4—C14	1.203 (3)	C10A—C11A	1.311 (14)
N4—C14	1.352 (3)	C10A—H10C	0.9700
N4—C11B	1.466 (13)	C10A—H10D	0.9700
N4—C12A	1.488 (5)	C11A—H11C	0.9700
N4—C12B	1.522 (8)	C11A—H11D	0.9700
N4—C11A	1.525 (12)	C13A—H13C	0.9700
N3—C9	1.360 (3)	C13A—H13D	0.9700
N3—C13A	1.396 (13)	C9—N2	1.313 (3)
N3—C10A	1.464 (6)	C7—C6	1.384 (3)
N3—C10B	1.471 (10)	C7—C2	1.400 (3)
N3—C13B	1.55 (2)	C2—C3	1.371 (4)
C8—O2	1.346 (3)	C2—H2	0.9300
C8—C7	1.461 (4)	C3—C4	1.374 (4)
O2—N2	1.423 (3)	C16—H16A	0.9600
C17—C15	1.517 (4)	C16—H16B	0.9600
C17—H17A	0.9600	C16—H16C	0.9600
C17—H17B	0.9600	C18—H18A	0.9600
C17—H17C	0.9600	C18—H18B	0.9600
C15—C18	1.513 (4)	C18—H18C	0.9600
C15—C16	1.516 (4)	C6—C5	1.371 (4)
C13B—C12B	1.40 (2)	C6—H6	0.9300
C13B—H13A	0.9700	C5—C4	1.392 (4)
C13B—H13B	0.9700	C5—H5	0.9300
C12B—H12A	0.9700	C4—H4	0.9300
			
C14—O3—C15	120.64 (19)	H11A—C11B—H11B	106.6
C8—N1—C9	102.38 (19)	N4—C12A—C13A	106.2 (7)
C1—O1—C3	117.1 (2)	N4—C12A—H12C	110.5
C14—N4—C11B	118.3 (4)	C13A—C12A—H12C	110.5
C14—N4—C12A	121.0 (3)	N4—C12A—H12D	110.5
C11B—N4—C12A	107.1 (5)	C13A—C12A—H12D	110.5
C14—N4—C12B	124.7 (4)	H12C—C12A—H12D	108.7
C11B—N4—C12B	116.9 (5)	C11A—C10A—N3	120.6 (6)
C12A—N4—C12B	39.3 (4)	C11A—C10A—H10C	107.2
C14—N4—C11A	117.5 (5)	N3—C10A—H10C	107.2
C11B—N4—C11A	23.3 (7)	C11A—C10A—H10D	107.2
C12A—N4—C11A	119.0 (5)	N3—C10A—H10D	107.2
C12B—N4—C11A	112.1 (6)	H10C—C10A—H10D	106.8
C9—N3—C13A	119.0 (4)	C10A—C11A—N4	116.1 (7)
C9—N3—C10A	117.9 (3)	C10A—C11A—H11C	108.3
C13A—N3—C10A	112.6 (7)	N4—C11A—H11C	108.3
C9—N3—C10B	124.8 (5)	C10A—C11A—H11D	108.3
C13A—N3—C10B	116.2 (6)	N4—C11A—H11D	108.3
C10A—N3—C10B	34.1 (5)	H11C—C11A—H11D	107.4
C9—N3—C13B	120.7 (8)	N3—C13A—C12A	112.4 (7)
C13A—N3—C13B	13.8 (16)	N3—C13A—H13C	109.1
C10A—N3—C13B	117.7 (8)	C12A—C13A—H13C	109.1
C10B—N3—C13B	112.4 (10)	N3—C13A—H13D	109.1
N1—C8—O2	113.7 (2)	C12A—C13A—H13D	109.1
N1—C8—C7	128.1 (2)	H13C—C13A—H13D	107.9
O2—C8—C7	118.2 (2)	N2—C9—N3	123.0 (2)
C8—O2—N2	106.38 (18)	N2—C9—N1	115.3 (2)
C15—C17—H17A	109.5	N3—C9—N1	121.6 (2)
C15—C17—H17B	109.5	C6—C7—C2	119.5 (2)
H17A—C17—H17B	109.5	C6—C7—C8	120.1 (2)
C15—C17—H17C	109.5	C2—C7—C8	120.4 (2)
H17A—C17—H17C	109.5	C3—C2—C7	119.2 (2)
H17B—C17—H17C	109.5	C3—C2—H2	120.4
O3—C15—C18	102.0 (2)	C7—C2—H2	120.4
O3—C15—C16	110.1 (2)	C2—C3—C4	122.2 (2)
C18—C15—C16	111.2 (3)	C2—C3—O1	118.2 (2)
O3—C15—C17	109.9 (2)	C4—C3—O1	119.5 (2)
C18—C15—C17	111.0 (2)	F2—C1—F3	109.1 (3)
C16—C15—C17	112.2 (2)	F2—C1—F1	105.3 (4)
O4—C14—O3	125.4 (2)	F3—C1—F1	105.4 (3)
O4—C14—N4	123.3 (2)	F2—C1—O1	115.3 (3)
O3—C14—N4	111.3 (2)	F3—C1—O1	109.3 (3)
C12B—C13B—N3	109.2 (16)	F1—C1—O1	111.8 (3)
C12B—C13B—H13A	109.8	C15—C16—H16A	109.5
N3—C13B—H13A	109.8	C15—C16—H16B	109.5
C12B—C13B—H13B	109.8	H16A—C16—H16B	109.5
N3—C13B—H13B	109.8	C15—C16—H16C	109.5
H13A—C13B—H13B	108.3	H16A—C16—H16C	109.5
C13B—C12B—N4	110.6 (13)	H16B—C16—H16C	109.5
C13B—C12B—H12A	109.5	C15—C18—H18A	109.5
N4—C12B—H12A	109.5	C15—C18—H18B	109.5
C13B—C12B—H12B	109.5	H18A—C18—H18B	109.5
N4—C12B—H12B	109.5	C15—C18—H18C	109.5
H12A—C12B—H12B	108.1	H18A—C18—H18C	109.5
C11B—C10B—N3	122.9 (9)	H18B—C18—H18C	109.5
C11B—C10B—H10A	106.6	C9—N2—O2	102.18 (19)
N3—C10B—H10A	106.6	C5—C6—C7	119.9 (2)
C11B—C10B—H10B	106.6	C5—C6—H6	120.1
N3—C10B—H10B	106.6	C7—C6—H6	120.1
H10A—C10B—H10B	106.6	C6—C5—C4	121.4 (3)
C10B—C11B—N4	122.7 (7)	C6—C5—H5	119.3
C10B—C11B—H11A	106.7	C4—C5—H5	119.3
N4—C11B—H11A	106.7	C3—C4—C5	117.9 (3)
C10B—C11B—H11B	106.7	C3—C4—H4	121.0
N4—C11B—H11B	106.7	C5—C4—H4	121.0
			
C9—N1—C8—O2	−0.7 (3)	N3—C10A—C11A—N4	23.9 (15)
C9—N1—C8—C7	178.7 (3)	C14—N4—C11A—C10A	173.0 (8)
N1—C8—O2—N2	0.1 (3)	C11B—N4—C11A—C10A	−88.8 (19)
C7—C8—O2—N2	−179.4 (2)	C12A—N4—C11A—C10A	−24.8 (13)
C14—O3—C15—C18	−179.3 (2)	C12B—N4—C11A—C10A	18.3 (13)
C14—O3—C15—C16	62.6 (3)	C9—N3—C13A—C12A	−156.9 (8)
C14—O3—C15—C17	−61.5 (3)	C10A—N3—C13A—C12A	59.0 (15)
C15—O3—C14—O4	2.3 (4)	C10B—N3—C13A—C12A	21.6 (19)
C15—O3—C14—N4	−176.4 (2)	C13B—N3—C13A—C12A	−56 (4)
C11B—N4—C14—O4	−20.6 (7)	N4—C12A—C13A—N3	−56.6 (15)
C12A—N4—C14—O4	−156.0 (6)	C13A—N3—C9—N2	8.0 (10)
C12B—N4—C14—O4	157.0 (9)	C10A—N3—C9—N2	150.3 (5)
C11A—N4—C14—O4	5.9 (8)	C10B—N3—C9—N2	−170.3 (9)
C11B—N4—C14—O3	158.2 (6)	C13B—N3—C9—N2	−7.8 (12)
C12A—N4—C14—O3	22.7 (6)	C13A—N3—C9—N1	−171.5 (9)
C12B—N4—C14—O3	−24.3 (10)	C10A—N3—C9—N1	−29.2 (6)
C11A—N4—C14—O3	−175.4 (7)	C10B—N3—C9—N1	10.2 (10)
C9—N3—C13B—C12B	144.2 (13)	C13B—N3—C9—N1	172.6 (12)
C13A—N3—C13B—C12B	58 (4)	C8—N1—C9—N2	1.2 (3)
C10A—N3—C13B—C12B	−14 (2)	C8—N1—C9—N3	−179.2 (3)
C10B—N3—C13B—C12B	−51 (2)	N1—C8—C7—C6	−5.7 (4)
N3—C13B—C12B—N4	55 (2)	O2—C8—C7—C6	173.7 (2)
C14—N4—C12B—C13B	147.2 (14)	N1—C8—C7—C2	174.9 (3)
C11B—N4—C12B—C13B	−35.2 (19)	O2—C8—C7—C2	−5.7 (4)
C12A—N4—C12B—C13B	49.1 (16)	C6—C7—C2—C3	−1.4 (4)
C11A—N4—C12B—C13B	−60.3 (18)	C8—C7—C2—C3	178.0 (2)
C9—N3—C10B—C11B	−171.3 (12)	C7—C2—C3—C4	0.0 (4)
C13A—N3—C10B—C11B	10 (2)	C7—C2—C3—O1	−176.6 (2)
C10A—N3—C10B—C11B	−81.8 (16)	C1—O1—C3—C2	−96.5 (3)
C13B—N3—C10B—C11B	25 (2)	C1—O1—C3—C4	86.9 (3)
N3—C10B—C11B—N4	−4 (2)	C3—O1—C1—F2	−62.4 (4)
C14—N4—C11B—C10B	−174.7 (12)	C3—O1—C1—F3	174.2 (2)
C12A—N4—C11B—C10B	−33.7 (15)	C3—O1—C1—F1	57.9 (4)
C12B—N4—C11B—C10B	7.5 (18)	N3—C9—N2—O2	179.3 (3)
C11A—N4—C11B—C10B	91 (2)	N1—C9—N2—O2	−1.1 (3)
C14—N4—C12A—C13A	−159.4 (7)	C8—O2—N2—C9	0.6 (3)
C11B—N4—C12A—C13A	60.8 (9)	C2—C7—C6—C5	2.4 (4)
C12B—N4—C12A—C13A	−51.0 (9)	C8—C7—C6—C5	−177.0 (3)
C11A—N4—C12A—C13A	38.9 (11)	C7—C6—C5—C4	−2.0 (5)
C9—N3—C10A—C11A	173.1 (9)	C2—C3—C4—C5	0.5 (4)
C13A—N3—C10A—C11A	−42.4 (13)	O1—C3—C4—C5	177.0 (3)
C10B—N3—C10A—C11A	61.3 (11)	C6—C5—C4—C3	0.6 (5)
C13B—N3—C10A—C11A	−28.1 (16)		

### Hydrogen-bond geometry (Å, º)

**Table:**

*D*—H···*A*	*D*—H	H···*A*	*D*···*A*	*D*—H···*A*
C17—H17*A*···O4^i^	0.96	2.56	3.393 (3)	145
C10*A*—H10*C*···O4^ii^	0.97	2.46	3.413 (10)	167

Symmetry codes: (i) *x*+1, *y*, *z*; (ii) −*x*+2, −*y*+1, −*z*+1.
